# The Influence of Interaction between Cadmium with 17β-Estradiol, 2-Methoxyestradiol and 16α-Hydroxyestrone on Viability and p-Glycoprotein in Ovarian Cancer Cell Line

**DOI:** 10.3390/ijms23052628

**Published:** 2022-02-27

**Authors:** Ewa Sawicka, Jolanta Saczko, Julita Kulbacka, Martyna Szydełko, Beata Szymańska, Agnieszka Piwowar

**Affiliations:** 1 Department of Toxicology, Faculty of Pharmacy, Wroclaw Medical University, Borowska 211, 50-556 Wroclaw, Poland; beata.szymanska@umw.edu.pl (B.S.); agnieszka.piwowar@umw.edu.pl (A.P.); 2 Department of Molecular and Cellular Biology, Faculty of Pharmacy, Wroclaw Medical University, Borowska 211A, 50-556 Wroclaw, Poland; jolanta.saczko@umw.edu.pl (J.S.); julita.kulbacka@umw.edu.pl (J.K.); 3 Students’ Scientific Society, Department of Toxicology, Faculty of Pharmacy, Wroclaw Medical University, Borowska 211, 50-556 Wroclaw, Poland; martyna.szydelko@gmail.com

**Keywords:** ovarian cancer, cadmium, 17β-estradiol, 2-methoxyestradiol, 16α-hydroxyestrone, P-glycoprotein

## Abstract

Occupational and environmental exposure to xenoestrogens, a subgroup of endocrine disruptors (EDCs), can affect the endocrine system and increase the risk of cancer, primarily the hormone-dependent kind. This type of cancer includes ovarian cancer, which is the leading cause of death from gynecological tumors. The aim of this study was to assess the role of 17β-estradiol and its metabolites: 2-MeOE2, 16α-OHE1 in exposure to the metalloestrogen cadmium. The effect of interactions of cadmium with estrogens on the viability of cells in malignant ovarian cancer cells SKOV-3 was investigated, both in simultaneous action and in the pre-incubation model. There are no known interactions between estrogens and cadmium in ovarian cancer cells. Due to the frequent occurrence of multidrug resistance (MDR) in ovarian cancer, the effects of estrogens and cadmium on MDR in SKOV-3, measured as P-glycoprotein (P-gp), were assessed. An interaction study showed that E2 had an antagonistic effect on cadmium-induced cell damage, while 2-MeOE2 showed less of a protective effect in combination with CdCl_2_ than E2. There were two types of interaction: toxic synergism and beneficial antagonism. E2 and cadmium increased P-gp expression in SKOV-3 cells, while 2-MeOE2 decreased P-gp expression to a potentially beneficial effect on MDR prevention. The obtained results constitute an interesting starting point for further research in the field of interactions between estrogens and xenoestrogens in ovarian cancer.

## 1. Introduction

Cadmium is a highly toxic non-essential transition metal that constitutes an important potential health risk to the environment [1]. Multiple mechanisms potentially relate cadmium to cancer; these include oxidative stress or inflammation, interference with DNA repair and changes of DNA methylation. More importantly in respect to hormone-related cancers, there is evidence that cadmium may act on estrogenic signaling pathways, resulting, among others, in the proliferation of breast cancer cells in vitro [2,3]. The literature data on the effect of cadmium on breast cancer are numerous, but there is little data on ovarian cancer (OCa). This study analyzes the available data from the Third National Health and Nutrition Examination Survey, which suggests a link between exposure to cadmium and increased mortality from ovarian cancer [4]. Moreover, García-Perez et al. [2015] revealed a statistically significant increase in the incidence of, and mortality from, ovarian cancer in the proximity of cadmium-emitting refineries [5]. 

Ovarian cancer is the leading cause of death from gynecologic malignancies. The highest rates of death (11.4 per 100,000 and 6.0 per 100,000, respectively) are seen in Eastern and Central Europe. Ovarian cancer is the second most common malignancy after breast cancer in women over the age of 40, particularly in developed countries [6,7]. Epidemiological studies have shown the importance of hormonal factors in the pathogenesis of OCa [8]. The relationship between estrogens and ovarian cancer is not so clear, but recent findings from a reanalysis of the epidemiological data suggest an elevated risk of ovarian cancer with estrogen and progesterone therapy or estrogen therapy only. The available data have shown that ovarian cancer cells are associated with a number of estrogen-regulated pathways, similarly to other hormone-dependent cancers [9]. An increase in 17β-estradiol (E2) is often observed in ovarian cancer. E2 is known to induce the proliferation of ovarian cancer cells by inhibiting cell-to-cell adhesion, which may cause metastasis [10,11].

The presence of xenoestrogens known as endocrine disrupting compounds (EDC) in occupational and environmental exposure may have an effect on the endocrine system and increase the risk of cancer, mainly hormone-dependent cancer [12]. Some xenobiotics can influence estrogen metabolism by inducing or inhibiting it. Many xenobiotics act as inducers or inhibitors of enzymes, including those involved in the metabolism of estrogens [13]. There are very little data in the literature regarding the influence of EDC on the pathogenesis of ovarian cancer. Park et al. [12] showed that activation of the estrogen response element (ERE) is involved in the proliferation of ovarian cancer cells. It is important to investigate the interactions of estrogens with carcinogens (e.g., cadmium), as estrogen itself is carcinogenic, and synergism with carcinogenic cadmium may potentially be harmful to health. Exposure to metals, including cadmium, is widespread, therefore the explanation of the role of these compounds in the development of hormone-dependent cancers may have important implications for disease prevention.

There has been increased interest in the role of estrogen metabolites in ovarian cancer pathogenesis, as metabolites can often be more toxic than estrogens themselves, e.g., causing DNA damage by creating mutagenic DNA adducts and generating oxygen free radicals. Estrogen metabolism proceeds through the hydroxylation of the A ring, and then 2- or 4-hydroxyl derivatives are formed. Hydroxylation of the D ring (at position 16α) may also take place [14]. For example, during estradiol biotransformation, 2-hydroxyestradiol (2-OHE2), 2-methoxyestradiol (2-MeOE2), 4-hydroxyestradiol (4-OHE2) or 16-hydroxyestrone (16α-OHE1) are present in the organism [15]. The anti-angiogenic properties of 2-MeOE2 have been demonstrated both in vivo and in vitro. It has also been shown that this metabolite has pro-apoptotic and antiproliferative properties in various cells in vitro [16]. In turn, the increased activity of 16a-hydroxylase, an enzyme involved in the formation of 16a-OHE1, was observed in the population of women with breast cancer [12]. Still, no specific studies have been performed on 16α-OHE1 in relation to the risk of ovarian cancer, and the potential role of estrogen metabolism in ovarian cancer has been found to be unclear.

Multidrug resistance (MDR) to chemotherapy is the major cause of treatment failure in ovarian cancer. MDR is a serious clinical problem that severely limits the success rate of chemotherapy in ovarian cancer treatment [17]. The earliest known mechanism of MDR is overexpression of the MDR1 gene and associated increased P-glycoprotein activity (P-gp), which is responsible for the active removal of many structurally unrelated cytostatics from tumor cells. P-glycoprotein is a transmembrane protein that belongs to subfamily B of ATP-binding cassette transporters (ABC transporters). Ovarian neoplasms exhibit secondary multi-drug resistance with the participation of P-gp under the influence of cytostatic treatment [18].

The aim of our work was to evaluate the role of 17β-estradiol and its metabolites: 2-MeOE2 and 16α-OHE1 upon exposure to the metalloestrogen cadmium, a toxic environmental agent. The interactions of cadmium with estrogens and their influence on cell viability in malignant cisplatin-resistant ovarian cancer cells SKOV-3 were examined both in simultaneous action and in the pre-incubation model. These interactions are still poorly understood. There are no data in the literature on the influence of cadmium with estrogens on the pathogenesis of ovarian cancer, therefore it seems interesting to investigate their relationship. In connection with the dual role of estrogens, the question arises whether estrogens play a protective role in exposure to cadmium or whether the toxic effect is increased and what kind of interactions occur. Additionally, based on the calculated coefficient Combination Index (CI), the type of interaction between estrogens and cadmium was assessed. Since ovarian cancer is one of many cancers associated with multi-drug resistance, we also studied the effects of estrogens and cadmium on glycoprotein expression. 

## 2. Results

### 2.1. Cytotoxicity Evaluation of Single Compounds

**Figure 1:**
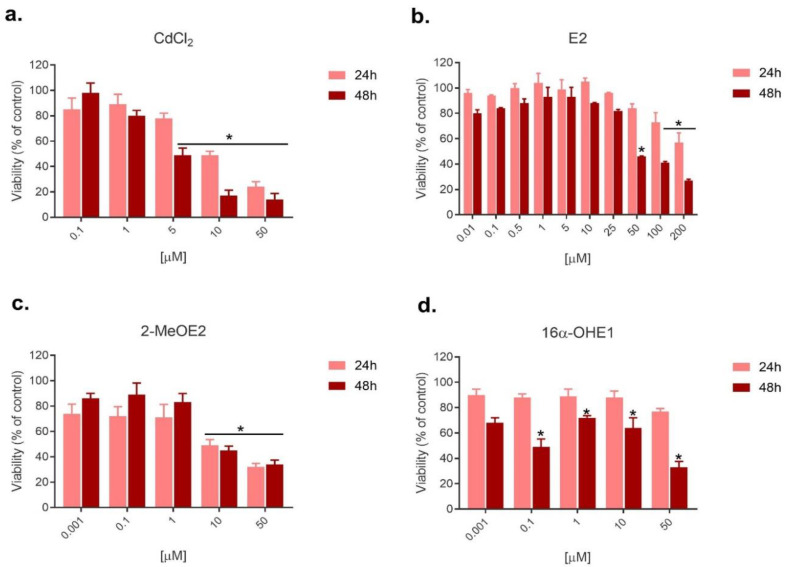
Changes in SKOV-3 viability after exposure to (**a**) CdCl_2_, (**b**) E2, (**c**) 2-MeOE2 and (**d**) 16α-OHE1, measured by MTT, after 24 h and 48 h (* statistically significant, *p* < 0.05).

The study of single compounds on SKOV-3 cell viability was conducted to establish the cytotoxicity profile of compounds and optimize their concentrations to estimate their combined effect. The MTT test was performed after 24 h and 48 h. Figure 1 shows SKOV-3 cell viability after the action of cadmium and estrogens. 

In the conducted research, we observed a statistically significant decrease in SKOV-3 viability after cadmium exposure. The cytotoxicity of this compound after 48 h was more clearly indicated than after 24h. A significant decrease in viability was observed starting from 5 μM, while the highest dose of Cd (50 μM) decreased viability to 14%. In high doses, 50–200 μM, E2 caused a statistically significant decrease in viability, especially after 48 h incubation, up to 46%, 41% and 27%, respectively. Estrogen metabolite exposure also caused a decrease in cell viability—2-MeOE2 led to a statistically significant decrease at two of its highest doses: 10 and 50 μM after both incubation times. Mainly longer 48 h exposure to 16α-OHE1 caused a statistically significant decrease in cell viability at 0.1 μM and 50 μM. The effect of longer incubation time was especially important for exposure to CdCl_2_, E2 and 16α-OHE1, where viability after 48 h was much lower (Figure 1).

### 2.2. Evaluation of the Simultaneous Effect of E2 and Its Metabolites with CdCl_2_—Interaction Study

**Figure 2:**
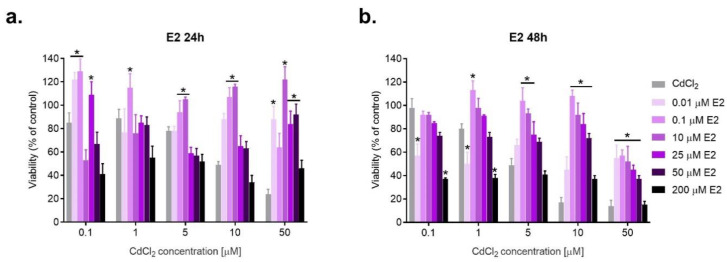
Changes in SKOV-3 viability after simultaneous effect of both E2 and CdCl_2_ measured by MTT after 24 h and 48 h against CdCl_2_ alone (* statistically significant, *p* < 0.05). The type of interaction between estrogens and cadmium, calculated on the basis of the Combination Index (CI), is given in Table 1, Table 2 and Table 3, respectively.

**Figure 3:**
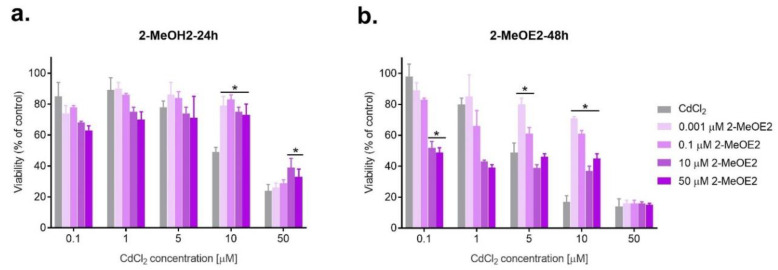
Changes in SKOV-3 viability after the simultaneous effect of both 2-MeOE2 and CdCl_2_ measured by MTT after 24 h and 48 h against CdCl_2_ alone (* statistically significant, *p* < 0.05).

**Figure 4:**
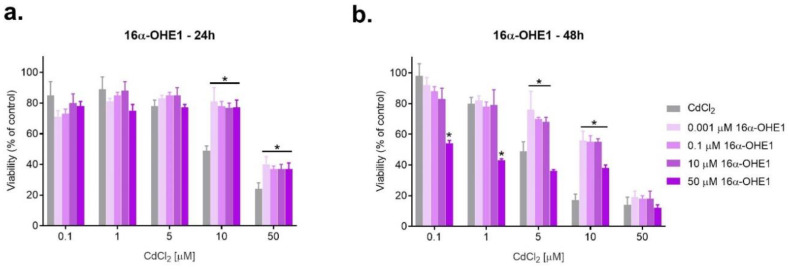
Changes in SKOV-3 viability after simultaneous effect of both 16α-OHE1 and CdCl_2_ measured by MTT after 24 h and 48 h against CdCl_2_ alone (* statistically significant, *p* < 0.05).

Based on the results obtained and the literature data for the estrogen–cadmium interaction studies, the following concentrations were selected: for E2—0.01, 0.1, 10, 25, 50, and 200 μM; for 2-MeOE2 and for 16α-OHE1—1 nM, 0.1, 10 and 50 μM. Moreover, the following doses were chosen for CdCl_2_: 0.1, 1, 5, 10 and 50 μM. (Figure 2, Figure 3 and Figure 4).

The nature of the interaction between E2 and CdCl_2_ depends to some extent on the time of incubation. Cases of synergism occur after 24 h, while after a longer incubation of 48 h, the dominant type of combined action is antagonism, especially after coexposure to lower doses of E2 (e.g., 0.1 and 10 μM), indicating a beneficial protective effect of E2 in SKOV-3 cells exposed to cadmium. Characteristically, high doses of E2 (200 μM) do not protect against cadmium action. The majority of cadmium interactions with this concentration of E2 are of toxic synergy, and the viability is even lower than in cells exposed to CdCl_2_ alone. E2 at 50 μM acted synergistically with lower doses of CdCl_2_. E2 (25 μM) caused an antagonistic effect with both low and high doses of CdCl_2_, showing a protective action on SKOV-3 cells. E2 (10 μM) was beneficial in cells exposed to Cd at 5–50 μM. The results are consistent for both incubation times, with a slightly predominant antagonistic effect after a longer, 48 h incubation (Figure 2, Table 1).

As is the case for exposure to E2, antagonism prevails in combined action with 2-MeOE2, with the beneficial effect of a shorter incubation time (24 h), especially for lower concentrations of cadmium. A statistically significant increase in viability was observed for 2-MeOE2 at a concentration of 0.001 μM (*p* < 0.05). The highest dose of the tested metabolite (50 μM) showed an antagonistic effect at most cadmium doses used in the experiment. However, 2-MeOE2 also had a protective effect, increasing the viability of ovarian cancer cells in a statistically significant manner, mainly with lower doses of metabolite (*p* < 0.05) (Table 2, Figure 3).

After incubation for 24 h, marked antagonism was observed when exposed to the combined action of cadmium at a concentration of 10 µM and 50 µM with all concentrations of 16α-OHE1 (*p* < 0.050). Synergism was also characteristic for the combined exposure of the highest concentration of the metabolite with low doses of cadmium (0.1 and 1.0 µM) (*p* < 0.05). Antagonism prevailed when SKOV-3 cells were exposed to a mixture of 16α-OHE1 and CdCl_2_ both after 24 h and 48 h of incubation (Figure 4, Table 3). 

### 2.3. Evaluation of the Effect of CdCl_2_ on SKOV-3 Cells after Pre-Incubation (24 h and 7 Days) with E2, 2-MeOE2 and 16α-OHE1

**Figure 5:**
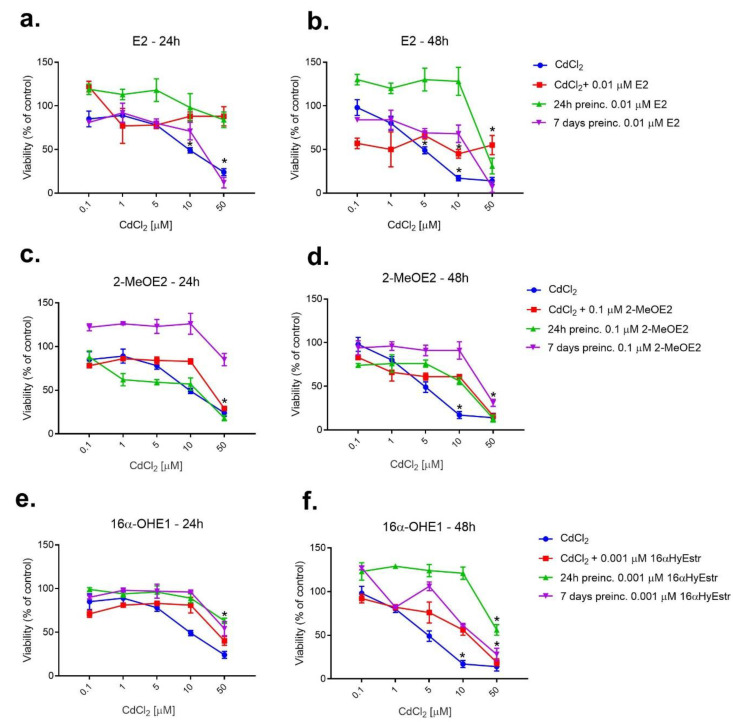
Changes in SKOV-3 viability after 24 h and 7 days of pre-incubation with E2 (**a**,**b**), 2-MeOE2 (**c**,**d**) and 16-OHE1 (**e**,**f**), and exposure to CdCl_2_ measured by MTT after 24 h and 48 h. The figure also shows the combined action of the interaction of the respective estrogen with CdCl_2_ (* statistically significant, *p* < 0.05).

In the following model of pre-incubation with estrogen, E2 or metabolites were added to cells at one concentration chosen during an earlier examination. The results are presented in Figure 5.

Based on preliminary research, non-toxic concentrations for 24 h and 7-day estrogen pre-incubation were selected: E2—0.01 μM; 2ME2—0.1 μM; 16α-OHE1—1 nM, to determine if estradiol or its metabolites could have a protective effect on SKOV-3 cells exposed to cadmium chloride. The effect of estrogens on cadmium-exposed SKOV-3 was examined by pre-incubating ovarian cancer cells with E2, then exposing them to CdCl_2_. The cells were pre-incubated with E2 at a concentration of 0.01 μM for 24 h or 7 days, and viability was measured after a further 24 h. When comparing 24 h and 7-day pre-incubation, many more beneficial effects were observed for 24 h pre-incubation (the highest cell viability). Seven-day pre-incubation was not as effective as 24 h pre-incubation, and in the case of high concentration of CdCl_2_ (50 μM), pre-incubation with E2 caused an increase in toxicity and up to 12% decrease in viability. The test conducted after a longer incubation of 48 h showed more pronounced differences in cell activity than after a shorter, 24 h incubation. Seven-day pre-incubation with 2-MeOE2 was the most beneficial. The protective effect of this metabolite on SKOV-3 cells pointed to a viability increase mainly after 24 h incubation. The cell viability test performed after 24 and after 48 h incubation indicated that 24 h pre-incubation resulted in no statistically significant change in viability in relation to cadmium alone or to the effect of this metabolite and metal co-exposure. Only the effect of the 7-day pre-incubation was very pronounced, especially during the shorter 24 h incubation. During the pre-incubation study with 16α-OHE1, it was noticed that the test performed after 24 h of incubation showed no significant differences in viability, however, both 24 h and 7-day pre-incubation increased cell viability compared to cadmium alone. Much more significant differences were visible when the test was performed after 48 h. In this case, a significant increase in viability was observed after 24 h of pre-incubation with 16α-OHE1 as well as following 7-day pre-incubation. This increase was also statistically significant. In the case of co-exposure to 16α-OHE1 and cadmium, the incubation time was observed to have a varied effect on the viability of SKOV-3. 

### 2.4. Results of the Immunocytochemical P-gp Assay on the SKOV-3 Line after Exposure to Estrogens and CdCl_2_

Table 4 shows the result of the action of E2 on P-gp expression. Cells exposed to E2 (0.01 μM) had very low expression of P-gp (3%), but at exposure to E2 (200 μM), the intensity of the reaction was very high (+++) and the percentage of stained cells was 90%. Under the influence of the lowest concentration of CdCl_2_ 0.1 μM, P-gp expression was 29% (+). The increase in metalloestrogen concentration induced significant protein expression and high intensity of reaction (++/+++; 82% positive cells) at 50 μM CdCl_2_. The expression after exposure to 2-MeOE2 was much lower than for E2 and CdCl_2_ (3–48%). For 16α-OHE1, the reaction occurred only in cells exposed to the two highest concentrations (10 μM and 50 μM) with high intensity of reaction (+++). The control sample was not exposed to the test compounds (Table 4). 

The combined protocols (E2 + CdCl_2_) enhanced the intensity of reaction, especially for E2 (10 μM). Exposure of cells to the action of E2 in conjunction with CdCl_2_ (50 μM) resulted in significant expression of P-gp in the range of 33–88%, depending on the concentration of E2. Higher steroid concentrations resulted in a greater intensity of reaction (++/+++) and a greater percentage of stained cells (Table 5).

Table 6 shows the combined effect of 2-MeOE2 + CdCl_2_ on P-gp expression. A different effect from E2 was observed, as the co-exposure of 2-MeOE2 + CdCl_2_ showed a very low percentage of stained cells (from 2% to 11%) as well as a low intensity of the reaction (+/++). All results were below the control. 

Table 7 shows the combined effect of 16α-OHE1 with CdCl_2_ on P-gp expression. This combined protocol indicated that the percentage of stained cells was low (1–26%), comparable to the effect of 2-MeOE2 in combination with CdCl_2_. The intensity of the reaction was mainly in the +/++ range, thus 16α-OHE_1_, like 2-MeOE2, did not increase P-gp expression, although expression was increased to a slightly greater extent in 16α-OHE_1_.

**Figure 6:**
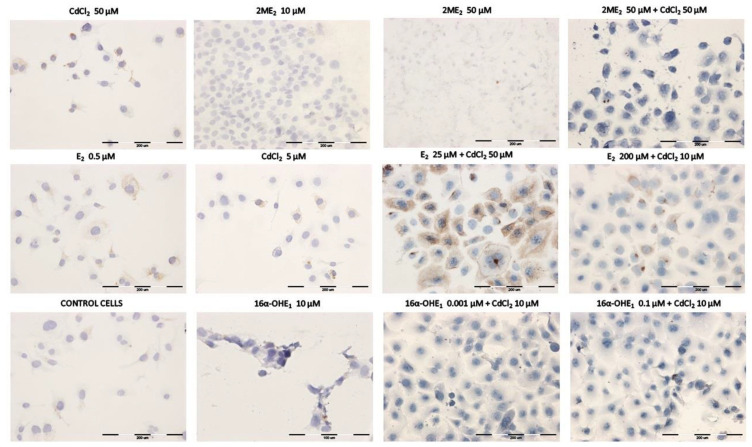
Immunoassayed reaction—exemplary expression results of P-glycoprotein in SKOV-3 ovarian cancer cells exposed to the single compounds and after exposure to the combined effects of estrogens and cadmium chloride.

The degree of P-gp expression in ovarian cancer cells under the influence of the tested compounds presented in the Table 4, Table 5, Table 6 and Table 7 reflects the microscopic results shown in Figure 6.

## 3. Discussion 

Compounds capable of affecting the human hormone balance by binding to the estrogen receptor are widespread in the environment. Cadmium is a xenoestrogen, does not fulfill biological functions, and is a common environmental pollutant, found among others in tobacco and cigarette smoke. It is a human carcinogen recognized by IARC and, according to the literature data, may play a significant role in the pathogenesis of various cancers [19]. The exact pathogenesis of OCa is still not fully understood. Increasing morbidity and the high mortality caused by OCa, estrogen-dependent cancer, justify research into the impact of environmental xenoestrogens on estrogen-dependent processes [4]. Estrogens stimulate cell proliferation and division, showing indirect action through metabolites that may have a protective or pro-carcinogenic effect, depending on the biotransformation pathway. While 2-MeOE2 is a metabolite of E2 with recognized antitumor activity, 16α-OHE1 is increasingly considered a compound with potential carcinogenic potential [15,16]. It seems important to understand the nature of the interaction between estrogens and cadmium, since estrogens are known for their protective functions and, on the other hand, the ability to induce carcinogenesis. Therefore, it is of interest whether estrogens have a protective effect on cells exposed to cadmium or if toxic synergy of action occurs.

An important aspect of many cancerous diseases, including ovarian cancer, is multi-drug resistance. Many mechanisms are responsible for the formation of MDR; one of the best-known is the overexpression of the membrane transporter P-glycoprotein. Many tissues and organs are characterized by a high physiological concentration of this protein, which is responsible for removing harmful xenobiotics outside the cells. Secondary multi-drug resistance with the participation of P-gp leads to the development of ovarian cancer. An excessive amount of this protein can be considered a multi-drug resistance marker [20]. In our examination, an attempt was made to assess whether estrogens may influence the expression of P-gp in ovarian carcinoma cells exposed to cadmium and, hence, to multi-drug resistance. Currently, there is no research in the literature on this subject, which is why studies carried out as part of this work have an element of novelty. 

The first stage of the study was the assessment of estrogens and cadmium toxicity on the SKOV-3 line, measured by the MTT test; the second stage involved a determination of P-gp expression under the influence of the tested compounds, measured by immunocytochemistry. The first stage of the study concerned the cytotoxicity of individual compounds, and then the combined action of estrogens with cadmium chloride was evaluated by two schemes described in the Methods section. The adverse effect of E2 on cell viability was noticeable for its high doses (50–200 μM). The remaining E2 concentrations (0.01–25 μM) were non-toxic. Although the effects of E2 on breast cancer cell lines have been studied, there is little research concerning metabolites. For example, Nunes et al. [21] conducted studies on the MCF-7 breast cancer cell line, in which they showed that E2 (100 nM) increases cell proliferation and has a cytoprotective effect. However, Seeger et al. [22] showed that, at physiological concentrations (0.1–10 nM), E2 enhances proliferation and decreases apoptosis of OVCAR-3 lines. Studies by Li et al. [23] also showed the antiapoptotic effects and increased proliferation in the same line by E2 (0.01–100 nM). Meanwhile, 2-MeOE2 is considered to be a metabolite with anticancer activity. Research carried out by Ding et al. [24] showed that 2-MeOE2 in combination with carboplatin significantly inhibited growth and induced apoptosis of SKOV-3 cells, showing a beneficial effect and sensitizing cells to cytostatics. Studies conducted by Saczko [25] have also shown that modifying photodynamic therapy by adding 2-MeOE2 significantly increases the effectiveness of the reaction and may eliminate cells of clear cell ovarian cancer (OvBH-1) by apoptosis. As was shown in our experiments, 16α-OHE1 decreased cell viability, particularly after 48 h incubation. The effect of 16α-OHE1 was clearly marked for high concentrations of the metabolite. There are only single studies concerning the effect of 16α-OHE1 on ovarian cancer. An experiment carried out by Seeger et al. [22] indicates the anti-apoptotic and proliferative effect of 16α-OHE1 (0.01–10 nM) on ovarian cancer cells OVCAR-3.

There are many papers on the influence of cadmium on the pathogenesis of cancers. A study by Zhang et al. [26] showed that exposure to cadmium may increase the risk of prostate cancer. A meta-analysis conducted by Lin et al. [27] shows that cadmium in the diet may increase the risk of breast cancer, and, according to studies by Jabłońska et al. [28], there is a correlation between increased cadmium concentration and the occurrence of breast cancer. There are only single experiments on the influence of cadmium on ovarian cancer. As a metalloestrogen, cadmium can affect estrogen-dependent processes. The ovarian membrane is rich in estrogen receptors, and there is a reasonable fear that this metal can affect the processes of cancer. The pathogenesis of ovarian cancer is still unexplained, so any research in this direction is important and will be helpful. Our research showed that cadmium chloride caused significant toxic effects on SKOV-3 cells, especially marked for concentrations of 5, 10 and 50 μM after 48 h incubation. Cytotoxicity was dependent on the concentration of the metal, with an increase of the dose lowering viability, amounting to 49%, 17%, 14%, respectively. Studies of the combined action of E2 with CdCl_2_ showed mostly a favorable protective effect of E2 on cadmium-exposed SKOV-3 cells after 24 h incubation. Analysis of the interaction performed using the CompuSyn program confirms its antagonism. The adverse toxic synergism was noted when combining the highest tested concentration of E2 (200 μM) with CdCl_2_, especially after 24 h incubation. The combined effect of E2 and CdCl_2_ after 48 h was advantageous and more clearly marked than 24 h incubation. From the above data, it can be concluded that, as manifested by reduced viability, high E2 concentration is toxic to the cancer cell line, which could be advantageous from the point of view of anti-cancer therapy with higher than physiological doses of estradiol. On the other hand, low doses of estradiol are protective in exposure to xenobiotics, so the protective effect of E2 in cadmium exposure may be suspect. The beneficial effect of 2-MeOE2 in interaction with cadmium was observed when exposed to high concentrations of CdCl_2_ (10–50 μM), which correlated with results from the CompuSyn program, indicating favorable antagonism. At lower concentrations of CdCl_2_ (0.1–5 μM), there was synergy alternating with antagonism. The above results suggest that low, close to physiological (0.001 and 0.1 μM) concentrations of 2-MeOE2, especially at longer incubation, may protect cells exposed to toxic cadmium. In contrast, high concentrations of the metabolite are characterized by a large antiproliferative effect on ovarian cancer cells, which may be beneficial in anticancer therapy. After 24 h incubation, 16α-OHE1 in interaction with CdCl_2_ showed a beneficial effect in SKOV-3 cells exposed to high doses of CdCl_2_ (5–50 μM), which corresponded to the results obtained from the interaction analysis (antagonism). At lower doses of CdCl_2_, estrogen showed toxic synergy. The high concentration of 16α-OHE1 worked unfavorably in combination with Cd for SKOV-3 cells, reducing their activity. In the case of CdCl_2_ (5–10 μM), a beneficial antagonistic effect appeared. 

In the second stage, the evaluation of the effect of E2 pre-incubation on CdCl_2_-induced damage in SKOV-3 cells, a clear beneficial effect of 24 h pre-incubation was observed. E2 protected SKOV-3 cells in the whole range of Cd concentrations. Seven-day pre-incubation was not as beneficial. The obtained result may prove the protective effect of estradiol in the case of cadmium-induced cytotoxicity, 24 h pre-incubation being sufficient to obtain the optimal effect. Seven-day pre-incubation with 2-MeOE2 produced positive results over the entire CdCl_2_ concentration range, both after 24 and 48 h, while 24 h estrogen pre-incubation did not yield such beneficial effects. From the above studies, it can be concluded that 2-MeOE2 may have a protective effect on cadmium cell damage after the cells’ long-term contact with estrogen and shorter exposure to the metal.

The next conducted part of the study involved the estimation of P-gp expression responsible for multidrug resistance. SKOV-3 ovarian cancer cells are characterized by P-gp overexpression. SKOV-3 cells exposed to E2 showed an increased expression of P-gp. Therefore, E2 in high doses has a negative effect on the cells; it increases defense processes in the cancer cell and may increase its multidrug resistance. The effect of low E2 concentrations would be beneficial in therapy. Similarly, in the case of CdCl_2_, the amount of P-gp in cells increases with the concentration of the metal. Cancer cells respond to toxic cadmium by increasing the expression of P-gp. In the case of chemotherapy, this response would involve not only cadmium, but also anti-cancer drugs. It can be concluded that MDR may increase after exposure to cadmium, and higher contamination of the environment may negatively affect anticancer treatment. Meanwhile, 2-MeOE2 significantly lowered the expression of P-gp. This effect could be very beneficial in reducing multidrug resistance and sensitizing cancer cells to therapy. The second metabolite tested, 16α-OHE1, did not give such unambiguous results. While it seems that it can also reduce the expression of P-gp in tumor cells, it does so in a smaller range of concentrations. Using the immunocytochemistry assay, the expression of P-gp after the combined application of estrogens and CdCl_2_ was evaluated. The role of E2 in such exposure has been shown to be ambiguous. Low E2 concentrations produced a positive effect (0.01–0.1 μM), reducing the expression of P-gp in cells induced by low concentrations of CdCl_2_ (0.1–10 μM), which can be considered as a reduction in cell sensitivity to toxic compounds and a beneficial effect in the context of anticancer therapies. In exposure to CdCl_2_, 2ME2 still had a positive effect, because the expression of P-gp was significantly reduced, regardless of the concentration of CdCl_2_. The significant reduction in P-gp expression caused by 16α-OHE1 would suggest a beneficial effect of this metabolite on tumor cells treated with xenobiotics, including anticancer drugs. Scientific articles indicate that multi-drug resistance of tumors is a serious problem that is still under investigation. An experiment conducted by Bradley et al. [29] showed an increasing overexpression of P-gp in SKOV-3 cells due to increasing concentrations of vinca alkaloids. In turn, Yang et al. [30] observed that SKOV-3 cell lines were characterized by increased expression of P-gp under the influence of cisplatin. The studies also showed that verapamil sensitized the cells of the test line to doxorubicin. There are many studies on the breakdown of multidrug resistance to increase the effectiveness of anticancer therapies. A study by Patel et al. [31] showed that coadministration of the P-gp inhibitor and paclitaxel into the tumor made it sensitive to cytostatics, indicating a promising way to overcome MDR.

To summarize, E2 had no toxic effect on SKOV-3 in low concentrations, while high concentrations of this hormone (50–200 μM) were cytotoxic, which may prove beneficial from the point of view of anti-cancer therapy. In the dose range of 0.001–1 μM, 2-MeOE2 did not show any toxic effects, and at concentrations of 10–50 μM it significantly reduced the viability of SKOV-3. In contrast, 16α-OHE1 showed toxic effects after longer incubation. The interaction study demonstrated the antagonistic effect of E2 on cadmium-induced cell damage. Meanwhile, 2-MeOE2 showed a weaker protective effect in combination with CdCl_2_ than E2. There were two types of interactions: toxic synergism and favorable antagonism, depending on the dose of the metabolite. Considering the effect of estrogen pre-incubation, a protective effect of 24 h estradiol incubation was noticed, while for 2-MeOE2, a longer, 7-day pre-incubation period was more beneficial. E2 and cadmium caused increased expression of P-gp in SKOV-3 cells, i.e., increased MDR, which may negatively affect anticancer treatment. Meanwhile, 2-MeOE2 significantly reduced the expression of P-gp with a potentially beneficial effect in the prevention of MDR. With simultaneous exposure to estrogen and cadmium, SKOV-3 cells were characterized by decreased expression of P-gp. The obtained results constitute an interesting starting point for further research in this area and for the analysis of pathways involved in both tumor progression and cancer cell death as a result of the applied therapies.

## 4. Material and Methods

### 4.1. Cell Culture

The research was conducted on the ovarian cell line SKOV-3. The cell line was obtained from the Department of Immunology, Center of Biostructure Research at Medical University of Warsaw as part of scientific cooperation, purchased in ATCC, and validated negatively according to mycoplasma. Line authentication was performed using a PCR-based technique that compares multiple short tandem repeat (STR) markers between two or more cell genomes with MycoBlue (Vazyme). The cultures were maintained in 37 °C and high humidity in the Steri-Cult^®^ Automated CO_2_ Incubator (Thermo Scientific, Alab, Poland). Dulbecco′s Modified Eagle′s Medium (DMEM) with a glucose concentration of 4500 mg/L (Sigma-Aldrich, Poznań, Poland), supplemented with fetal bovine albumin (10%) (FBS, Sigma-Aldrich, Poland) and 1% antibiotic solution containing 10,000 units penicillin and 10 mg streptomycin/mL (Sigma Aldrich, St. Louis, MO, USA) were used as a culture medium. 

### 4.2. Compounds 

The estrogens E2, 2-MeOE2, 16α-OHE1 and cadmium chloride (CdCl_2_) were used as a source of cadmium ions for this study. The list of compounds was presented in Table 8. Estrogen solutions were prepared in 96% ethanol, while distilled water was used for CdCl_2_ (Table 8).

### 4.3. Cytotoxicity Assay of Individual Estrogens and CdCl_2_

Cell viability was evaluated by MTT assay according to the manufacturer protocol (Sigma-Aldrich). First, cells were incubated with estrogens (E2, 2-MeOE2, 16α-OHE1) or CdCl_2_ independently (separately) to evaluate the cytotoxicity of these compounds to SKOV-3 cells. The following concentrations of compounds were used: E2—0.01, 0.1, 0.5, 1.0, 5.0, 10.0, 25.0, 50, 100.0 and 200.0 µM; 2-MeOE2 and 16α-OHE1—0.001, 0.1, 1.0, 10.0, 50 µM; CdCl_2_—0.1, 1.0, 5.0, 10.0 and 50.0 µM. After separate exposure, concentrations were chosen for further studies of the combined effect of E2 or its metabolites with CdCl_2_ on the ovarian cancer cell line. To determine cytotoxicity, cells were seeded into 96-well culture plates at a concentration of 5 × 10^4^ cell/well (Nunc, NunclonTM Surface, Biokom, Poland) according to the previously described method [32]. All tests were performed in triplicates.

### 4.4. Combined Effect of Cadmium with Estrogens

In the first model of combined effect-simultaneous action, single estrogens and Cd ions were added to the cell culture simultaneously and incubated for 24 h and 48 h at 37 °C. The dose selection for the interaction study was dictated by the study of individual compounds: for E2—0.01, 0.1, 10.0, 25.0, 50.0 and 200 µM; for 2-MeOE2 and 16α-OHE1—0.001, 0.1, 10.0 and 50 µM; for Cd—0.1, 1.0, 5.0, 10.0 and 50.0 µM.

To estimate the type of interaction, the combination index (CI) was calculated by CompuSyn software. The CI was calculated for the IC50 obtained during an in vitro experiment (data not shown). The results were interpreted as follows: CI = 1 indicates the additive effect of the test substances on the viability of ovarian cancer cells; CI < 1 indicates synergy (S) between the compounds used for the study; while CI > 1 indicates the antagonism (A) that occurs between them [33]. 

In the model of pre-incubation, first estrogen was added in one selected dose: E2—0.01 µM; 2-MeOE2—0.1 µM; 16α-OHE1—0.001 µM. Next, CdCl_2_ was added in the following doses: 0.1, 1.0, 5.0, 10.0 and 50.0 μM. The number of cells used for all experiments was the same as for simultaneous action. In the first step, estrogen was added to the culture medium and the cells were pre-incubated in the culture flask for 24 h or 7 days (with a million cells per flask). Then, cells were seeded in 5 × 10^4^ cell/well assay plates and incubated for 24 h to stick the cells to the wells. The cadmium solution was then added to the culture medium in appropriate concentrations. The plates were incubated for 24 h or 48 h, respectively. Then, the cytotoxicity test was used. All tests were performed in triplicates.

### 4.5. P-gp Estimation—Immunocytochemical Staining

Additionally, the expression of P-gp was investigated in SKOV-3 ovarian cancer cells. This study assessed the expression of P-gp in SKOV-3 ovarian cancer cells exposed to the single compounds and after exposure to the combined effects of estrogens and cadmium chloride. Cells were plated on 10-well slides (Thermo Scientific, Waltham, MA, USA) and incubated for 24 h. The slides were then rinsed with PBS and fixed with 4% paraformaldehyde. The next step was to perform an immunocytochemical test with the Expose Mouse and Rabbit Specific HRP/DAB Detection IHC kit (Abcam, Waltham, MA, USA, ab80436). The kit contained the following reagents: Mouse Determination Reagent, HRP Conjugate, DAB Substrate, DAB Chromogen, Hydrogen Peroxide Block.

Briefly, after washing with PBS (3 × 5 min), peroxidase activity was blocked by 30 min incubation with 1% H_2_O_2_; then, samples were permeabilized by incubation with 1% Triton X-100 (Sigma, Poland) in PBS (LabEmpire, Rzeszów, Poland). The cells were then incubated with the selected antibodies overnight at 4 °C. Primary antibodies (diluted 1:200, purchased from Abcam, USA) were used: anti-Aquaporin 4 [4/18] antibody—mouse monoclonal IgG (Abcam, USA ab9512) and anti-VDAC1/Porin antibody—rabbit polyclonal IgG (ab34726, Abcam). Cells were incubated with a secondary horseradish peroxidase (HRP) conjugated antibody. The samples were then incubated with a mixture of diaminobenzidine-H2O2 to show the HRP marker and were counterstained with hematoxylin (Roth, Poland) for 3 min. After dehydration in a gradient of ethanol (Chempur, Rzeszów, Poland) and xylene (Chempur, Poland), the microscope slides were covered with DPX (Aqua-Med Zpam-Kolasa, Poland). A vertical microscope (Olympus BX53, Warszawa, Poland) was used for sampling. The number of stained cells was determined by counting 100 cells in 3 randomly selected fields. First, the staining of the cells was tested. The percentage of stained cells was shown in the table, after which the intensity of the staining was estimated. The intensity of the immunohistochemical staining was assessed as (-) negative (no reaction), (+) weak, (++) moderate and (+++) strong [34].

### 4.6. Statistical Analysis

All values were expressed as mean ± SD. The normality of the distribution was assessed by the Lilliefors test. Differences between groups were assessed by one-way analysis of variance (ANOVA), which compares three or more unmatched groups, based on the assumption that the populations are Gaussian. Analysis was performed using the GraphPad Prism 7 software (GraphPad Software—DMW Communication, San Diego, CA, USA, CA). Values of *p* < 0.05 were considered significant. Dose-effect analysis of the combination treatment was calculated using the combination index (CI) by CompuSyn software [33].

In conclusion, the protective effect of estrogens may play a significant role in the pathogenesis of OCa, especially in environmental exposure to cadmium compounds. According to the research conducted as part of this work, the examined compounds may show a positive effect on cells exposed to cadmium, protecting them from damage. On the other hand, given the results, hormones can reduce the effectiveness of chemotherapy. Similarly, in the case of MDR, the tested metabolites may have a positive effect on the effects of treatment, but there is also a risk of limiting the effect of estradiol in the presence of environmental pollutants, which undoubtedly include cadmium.

## Figures and Tables

**Table 1:** Type of interaction after the combined effect of E2 and CdCl_2_ on SKOV-3 cells in the MTT test after 24 h and 48 h of incubation, using the CompuSyn program (S, synergism; A, antagonism).

	CdCl_2_ [µM]									
	Incubation 24 h					Incubation 48 h				
E_2_ [µM]	0.1	1	5	10	50	0.1	1	5	10	50
0.01	A	S	A	A	A	S	S	A	A	A
0.1	A	A	A	A	A	A	A	A	A	A
10	S	S	A	A	A	A	A	A	A	A
25	A	A	S	A	A	A	A	A	A	A
50	S	A	S	A	A	A	A	A	A	A
200	S	S	S	S	A	S	S	S	A	A

**Table 2:** Type of interaction after the combined effect of 2-MeOE2 and CdCl_2_ on SKOV-3 cells in the MTT test after 24 h and 48 h of incubation, using the CompuSyn program (S, synergism; A, antagonism).

	CdCl_2_ [µM]									
	Incubation 24 h					Incubation 48 h				
2-MeOE2 [µM]	0.1	1	5	10	50	0.1	1	5	10	50
0.001	S	A	A	A	S	S	A	A	A	A
0.1	A	A	A	A	S	A	S	A	A	A
10	A	A	A	A	A	S	S	S	A	A
50	A	A	A	A	S	A	S	A	A	A

**Table 3:** Type of interaction after the combined effect of 16α-OHE1 and CdCl_2_ on SKOV-3 cells in the MTT test after 24 h and 48 h of incubation, using the CompuSyn program (S, synergism; A, antagonism).

	CdCl_2_ [µM]									
	Incubation 24 h					Incubation 48 h				
16α-OHE1 [µM]	0.1	1	5	10	50	0.1	1	5	10	50
0.001	S	A	A	A	A	A	A	A	A	A
0.1	S	A	A	A	A	A	A	A	A	A
10	S	A	A	A	A	A	A	A	A	A
50	S	S	A	A	A	A	S	S	A	A

**Table 4:** Positive grading quantification of immunocytochemical staining of P-gp in the SKOV-3 line under the influence of estrogens and CdCl_2_. The number of stained cells was determined by counting 100 cells in 3 randomly selected fields. The intensity of the immunohistochemical staining was assessed as (-) negative (no reaction), (+) weak, (++) moderate and (+++) strong.

Compound	% of Stained Cells	Intensity of Reaction
**CdCl_2_ [µM]**		
**0.1**	29%	+
**1**	30%	+/++
**5**	52%	++/+++
**10**	47%	+/++
**50**	82%	++/+++
**E2 [µM]**		
**0.01**	3%	++
**0.5**	52%	++
**5**	45%	+/++
**25**	74%	++/+++
**200**	90%	+++
**2-MeOE2 [µM]**		
**0.001**	14%	+/++
**0.1**	8%	+/+
**1**	6%	+/+
**10**	3%	++/+++
**50**	45%	++/+++
**16α-OHE1 [µM]**		
**0.001**	0%	-
**0.1**	0%	-
**1**	0%	-
**10**	44%	++/+++
**50**	6%	++/+++
**Control**	53%	++

**Table 5:** Positive grading quantification of immunocytochemical staining of P-gp in the SKOV-3 line under the influence of E_2_ with CdCl_2_. The number of stained cells was determined by counting 100 cells in 3 randomly selected fields. The intensity of the immunohistochemical staining was assessed as (-) negative (no reaction), (+) weak, (++) moderate and (+++) strong.

CdCl_2_ [µM]	% of Stained Cells	Intensity of Reaction	CdCl_2_ [µM]	% of Stained Cells	Intensity of Reaction
**E_2_ 0.01 µM**			**E_2_ 25 µM**		
**0.1**	21%	++	**0.1**	49%	++
**1**	19%	+/++	**1**	31%	++
**5**	18%	++	**5**	30%	++
**10**	27%	++/+++	**10**	36%	++/+++
**50**	52%	++/+++	**50**	88%	+++
**E_2_ 0.1 µM**			**E_2_ 50 µM**		
**0.1**	10%	++/+++	**0.1**	47%	++
**1**	10%	++/+++	**1**	28%	++
**5**	21%	++	**5**	19%	+/++
**10**	19%	++	**10**	37%	++
**50**	43%	++	**50**	26%	+/++
**E_2_ 10 µM**			**E_2_ 200 µM**		
**0,1**	32%	+++	**0,1**	17%	+
**1**	31%	+++	**1**	48%	++
**5**	46%	+++	**5**	43%	++
**10**	43%	+++	**10**	47%	++/+++
**50**	35%	+++	**50**	58%	+++
**Control**	33%	++/+++	**Control**	33%	++/+++

**Table 6:** Positive grading quantification of immunocytochemical staining of P-gp in the SKOV-3 line under the influence of 2-MeOE2 with CdCl_2_. The number of stained cells was determined by counting 100 cells in 3 randomly selected fields. The intensity of the immunohistochemical staining was assessed as (-) negative (no reaction), (+) weak, (++) moderate and (+++) strong.

CdCl_2_ [µM]	% of Stained Cells	Intensity of Reaction	CdCl_2_ [µM]	% of Stained Cells	Intensity of Reaction
**2-MeOE2 0.001 µM**			**2-MeOE2 10 µM**		
**0.1**	2%	+	**0.1**	2%	++
**1**	6%	+	**1**	4%	++
**5**	11%	+	**5**	7%	++
**10**	10%	+	**10**	2%	++
**50**	9%	+	**50**	11%	++
**2-MeOE2 0.1 µM**			**2-MeOE2 50 µM**		
**0.1**	10%	++	**0.1**	5%	++
**1**	10%	++	**1**	3%	++
**5**	5%	++	**5**	5%	+
**10**	0%	-	**10**	3%	++
**50**	0%	-	**50**	9%	++
**Control**	33%	++/+++	**Control**	33%	++/+++

**Table 7:** Positive grading quantification of immunocytochemical staining of P-gp in the SKOV-3 line under the influence of 16α-OHE1 with CdCl_2_. The number of stained cells was determined by counting 100 cells in 3 randomly selected fields. The intensity of the immunohistochemical staining was assessed as (-) negative (no reaction), (+) weak, (++) moderate and (+++) strong.

CdCl_2_ [µM]	% of Stained Cells	Intensity of Reaction
**16α-OHE1** **0.001 µM**		
**0.1**	1%	+
**1**	20%	++
**5**	25%	+/++
**10**	11%	++
**50**	14%	+/++
**16α-OHE1** **0.1 µM**		
**0.1**	0%	-
**1**	0%	-
**5**	16%	+++
**10**	12%	++/+++
**50**	21%	++/+++
**16α-OHE1** **10 µM**		
**0.1**	11%	++
**1**	10%	++/+++
**5**	14%	++
**10**	10%	+
**50**	26%	+
**Control**	33%	++/+++

**Table 8:** List of compounds used in the experiments.

Name of Compound and Manufacturer	Structure	Molecular Weight	Stock Solution
**E2** SIGMA-ALRDICH	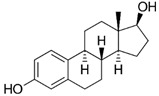	272.38 g/mol	**50 mM** ethanol solution
**2-MeOE2** SIGMA-ALDRICH	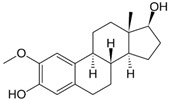	302.41 g/mol	**10 mM** ethanol solution
**16α-OHE1** STERALOIDS	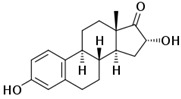	286.40 g/mol	**10 mM** ethanol solution
**Cadmium chloride** SIGMA-ALDRICH	CdCl_2_	183.32 g/mol	**50 mM** water solution
